# Relationship between tumor infiltrating lymphocytes and hormone-sensitive (breast and prostate) cancer survival: cases received at Yaoundé General Hospital (Cameroon) from 2019 to 2023

**DOI:** 10.11604/pamj.2025.50.78.43588

**Published:** 2025-03-18

**Authors:** Stéphane Zingue, Etienne Okobalemba Atenguena, Kevine Ghubap Makamte, Danielle Ingrid Tekam Maliedje, Laure Leka Zingue, Manuella Mayemi, Estelle Alida Ngne Mbopda, Zacharie Sando

**Affiliations:** 1 Basic and Clinical Cancer Research Unit, Department of Pharmacotoxicology and Pharmacokinetics, Faculty of Medicine and Biomedical Sciences, P.O. Box 1364, Yaoundé, Cameroon,; 2 School of Health Sciences, Catholic University of Central Africa, Yaoundé, Cameroon,; 3 Oncology Division, Yaoundé General Hospital, Yaoundé, Cameroon,; 4 Department of Internal Medicine, Faculty of Medicine and Biomedical Sciences, University of Yaoundé I, P.O. Box 1364, Yaoundé, Cameroon,; 5 Department of Morphological Sciences and Pathological Anatomy, Faculty of Medicine and Biomedical Sciences, University of Yaoundé I, P.O. Box 1364, Yaoundé, Cameroon

**Keywords:** Tumor-infiltrating lymphocytes, breast cancer, prostate cancer, hormone-dependent cancer

## Abstract

**Introduction:**

cancer ranks as the second leading cause of mortality worldwide, manifesting in diverse forms and impacting various tissues, notably prostate cancer (PCa) in men and breast cancer (BCa) in women. Cancer is a malignant tumor that can induce an immune response. During this reaction, immune cells are produced which are responsible for ridding the body of senescent and/or tumor cells, and constitute tumor effectors. Studies have shown that the presence of tumor-infiltrating lymphocytes (TILs) is a good prognostic marker for assessing survival in cancer patients; however, no similar data have yet been published in Cameroon.

**Methods:**

the aim of this study was to investigate the relationship between TILs and the survival of breast and prostate cancer patients at Yaoundé General Hospital (YGH) between 2019 and 2023. For this, a retrospective and cross-sectional study was undertaken at the Oncology Department and the Anatomo-Cytopathology Laboratory of YGH. A consecutive non-probability sampling (from August to October 2023) of 212 breast cancer patients and 89 prostate cancer patients who agreed to participate in the study constituted our sample. Variables were survival, TILs, sociodemographic, clinical, and pathological characteristics of patients. The TILs were estimated after Hematoxylin-Eosin (HE) staining and staged as low, middle and high TILs rate. The variables were sociodemographic characteristics (age, education level, religion, marital status, ethnolinguistic area, and menopause); clinical characteristics (affected breast, histological type, histological grade, type of treatment, AJCC stage, consistency of discovery); different grades of TILs. Furthermore, the correlation between TILs and chemotherapy as well as TILs and survival were analyzed. The obtained data were analyzed using Microsoft Excel, Epi-info 7 and SPSS software and statistical significance was considered at p< 0.05. Furthermore, the Cox regression analysis to identify factors associated with survival was performed.

**Results:**

the mean age was 43.56 ± 11.90 years for BCa with stage II and SBR grade II being the most common with proportions of 48.34% and 54.72%, respectively (n= 212). While in PCa, the mean age was 75 to 84 years (34.83%, n= 89) with stage II being the most frequent. Microscopic analysis of breast tissues revealed four TILs grades: absent (17.45%), low (18.40%), intermediate (27.83%) and high TIL grade (36.32%), while in PCa the most represented TILs grade was intermediate (31.46%). In BCa the intermediate TILs grade was positively associated with a good response to chemotherapy (p< 0.05). The overall 5-year survival in this study was around 44% for BCa, whereas it was 42 months for those with infiltration in PCa. No positive correlation was found between TILs grade and survival in BCa patients (p= 0.45), while in PCa, it was found that TILs are significantly correlated (p =0.016) with survival. Patients with prostate cancer who had not taken treatment had a 7.23 greater risk of death (HR: 7.23, 95% CI 1.21-53.14; p= 0.049) than those who had.

**Conclusion:**

no positive association was observed between TILs as graded and 5-year survival in BCa patients; however, it may have prognostic value in PCa patients. Further studies are encouraged to investigate the association between BCa molecular groups and TILs.

## Introduction

Cancer is a disease characterized by the uncontrolled proliferation of abnormal cells in the body, comprising different subgroups depending on their morphological, histopathological, or biochemical characteristics [1]. It is a truly global threat, with an estimated 19.3 million new cases and nearly 10 million cancer deaths in 2020, out of which BCa accounted for 2.3 million new cases and PCa with 1.4 million new cases and 375,000 deaths [2,3]. In sub-Saharan Africa, particularly in Cameroon, the prevalence of breast cancer is escalating, with 4,170 new cases (20.1%) and almost 2,108 deaths recorded in Cameroon in 2020 [4]. Concerning PCa, in Africa alone, there were 26,392 new cases resulting in approximately 7,709 fatalities [5]. Factors such as aging, population growth, poor dietary habits (obesity), socioeconomic conditions, reproductive factors, genetic factors, and heightened cancer risk factors contribute to the rapid rise in cancer incidence worldwide [1].

Hormone-sensitive cancers are often associated with good survival due to the large number of treatment strategies available. According to Ngowa *et al*. [6], the 5-year survival rate for BCa in Cameroon was 30% between 1997 and 2005, compared to 90% in some developed countries like France. However, efforts are underway to improve survival by increasing access to healthcare and strengthening early detection programs. Thus, between 2010 and 2015, the 5-year survival of BCa in Cameroonian patients increased to 43.3% [7]. Numerous studies have shown that early tumor diagnosis enables effective management and increases the overall survival of cancer patients [8]. Characterization of the tumor's immune microenvironment has led to the identification of new prognostic and predictive biomarkers and the development of new therapeutic targets and strategies [9].

Tumor-infiltrating lymphocytes (TILs) are crucial players in the immune response to cancer, the course of which also depends on other cells in the microenvironment. Tumor-infiltrating lymphocytes are a heterogeneous group of cells comprising B cells, T cells, and natural killer (NK) cells [9]. They are characterized as TILs when they migrate from the bloodstream to a tumor and are found both in the tumor itself and in the stroma surrounding it [10]. They are considered a good prognostic marker for cancers as they can indicate the body's immune response to the tumor and are responsible for the development of antitumor immune responses which can detect tumor antigens and destroy them [11]. Despite this, no studies correlating TILs with the survival of patients with hormone-sensitive (breast and prostate) cancers have been conducted in Cameroon. The purpose of this study was therefore to evaluate the relationship between TILs and survival in patients with breast and prostate cancers over the last 5 years at YGH.

## Methods

**Study design and setting:** this study was a retrospective cross-sectional study conducted in the Oncology Division and Anatomo-Cytopathology Laboratory of the YGH. Yaoundé General Hospital is one of the most specialized cancer treatment hospitals in Cameroon. It has numerous departments specializing in cancer treatment, including medical oncology, pathological anatomy, nuclear medicine, gynecology, and surgery. Patients with cancer treated at the YGH were often referred by other health establishments and came from all regions of the country. All patients diagnosed with breast and prostate cancers registered at the YGH during the study period (2019-2023) were considered. A total of 301 medical records were included in this study after successive and consecutive recruitment of all patients' medical records found i.e. 212 for breast cancer and 89 for prostate cancer. Medical records excluded were those of patients from 2019-2023 who had not given informed consent, or whose medical records could not be found or were incomplete, as well as patients who did not have biopsy samples or surgical specimens available in the Anatomo-Cytopathology Department.

**Figure 1:**
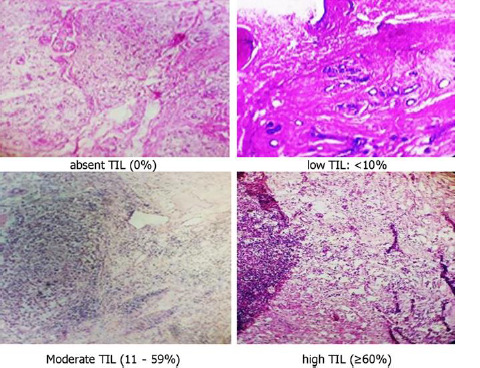
grade of tumor-infiltrating lymphocytes (TILs) on microscopy on a breast tumor

**Study population:** digital medical records of patients with hormone-sensitive cancers followed at the YGH between 2019 and 2023 were used. All medical records of patients included in this study during the 5-year study period were reviewed. Data collected and analyzed included: sociodemographic characteristics (age, education level, religion, marital status, ethnolinguistic area, and menopause); clinical characteristics (affected breast, histological type, histological grade, type of treatment, AJCC stage, consistency of discovery); different grades of TILs. Sections and biopsies were analysed after staining with hematoxylin-eosin (H&E 200x and 400x) under a light microscope by a pathologist. The TILs evaluated were those located in the tumor stroma zone. Using a light microscope, the rate of lymphocytic infiltration in tumor and peritumoral tissue was determined as a percentage in the observed area and defined as follows: absent (0%), low TIL (≤10%); intermediate TIL (10-59%) and high TIL (≥60%) following the international recommendation of Salgado *et al*. [12] (Figure 1). Only stromal TILs have been considered in this study; TILs present in areas with grinding artefacts, necrosis, and inflammation around biopsy sites were not taken into account.

**Data collection:** a 5-year survival was calculated as the time elapsed between the date of cancer diagnosis and the date of any cause, or the date of loss of sight, or the date of the last follow-up. Overall survival at 5 years was calculated by the Kaplan Meier method using the SPSS “Statistical Package for Social Sciences” software. Once data collection was complete, we checked the completeness and plausibility of the information obtained on the collection forms. We then created a data entry mask using Excel 2010.

**Bias:** all medical records of breast and prostate cancer patients arriving at the hospital during the study period were included to avoid bias.

**Data analysis:** once our data matrix had been obtained, it was transferred to Epi-Info 7 software for statistical analysis. We report descriptive categorical data with percentage and descriptive numerical data with mean and standard deviation. A p-value below 0.05 was considered statistically significant. Survival was calculated by the Kaplan-Meier method using SPSS software, and hazard ratio were calculated by cox proportional Hazard regression with 95% confidence interval.

**Ethical consideration:** study protocols were approved by the institutional ethics committee of the Catholic University of Central Africa, Cameroon. Patient consent was required for this cross-sectional study.

## Results

**General characteristics of the study population:** during the study period, 260 patients with breast cancer were recruited, 48 of whom were excluded (5 who failed to give informed consent, 18 whose paraffin blocks were unusable and 25 with incomplete medical records) affording a final sample size of 212. In the same period, 130 prostate cancer patients were diagnosed and managed at the YGH. Only 115 files were available, of which 99 patients were included. However, 10 patients had unavailable biopsies, giving us a final sample size of 89. The results presented for BCa patients in Table 1 show that the mean age was 43.56 ± 11.90 years, with a minimum of 20 and a maximum of 79 years old. The majority (43.87%; 93/212) of BCa patients were between 35 and 50 years old and had a primary school education, with a proportion of 30.66% (65 out of 212). The Grassfields, with a proportion of 41.21% (75 out of 182), were the most represented ethnocultural area, and most patients (90/212; 42.5%) belonged to the Catholic religion. Table 2, which presents the characteristics of the PCa patients, shows that 31 out of 89 (34.83%) of this population were between 75 and 84 years of age, and 37 out of 89 (41.57%) of them belonged to the Catholic religion. Married people were the most represented with 40 out of 89 (44.94%), and the majority of the population belonged to the Fang-Beti ethnolinguistic area with 43/89 (48.31%), and 38/89 (42.70%) were from the Grassfields.

**Table 1:** distribution of socio-demographic characteristics of patients with breast cancer

Variables	Frequency	Percentage (%)
**Age**		
20-35	55	25.9
35-50	93	43.9
50-65	54	25.5
65-79	10	4.7
**Level of education**		
Not enrolled	56	26.4
Primary	65	30.7
Secondary	60	28.3
Superior	31	14.6
**Ethnocultural area (n=182)**		
Grassfield	75	41.2
Fang Béti	52	28.6
Sawa	31	17.0
Sudan-Sahel	21	11.5
Foreign	3	1.6
**Marital status**		
Married	90	42.5
Widow	51	24.1
Single	43	20.3
Divorced	28	13.2
**Religion**		
Catholic	90	42.5
Protestant	46	21.7
Other	41	19.3
Muslim woman	20	9.4
Animist/traditional	15	7.1
**Menopause**		
Yes	165	77.8
No	47	22.2
**Total**	212	100

**Table 2:** distribution of socio-demographic characteristics of patients with prostate cancer

Variables	Frequency	Percentage (%)
**Age**		
<65	21	23.6
65-74	27	30.3
75-84	31	34.8
85+	10	11.2
**Level of education**		
Not enrolled	35	39.3
Primary	31	34.8
Secondary	10	11.2
Superior	13	14.6
**Ethnocultural area**		
Fang beti	43	48.3
Grassfield	38	42.7
Sahelian Sudan	8	9.0
**Marital status**		
Single	2	2.2
Divorced	8	9.0
Married	40	44.9
Widower	39	43.8
**Religion**		
Animist/traditional	16	18.0
Catholic	37	41.6
Muslim	5	5.62
Protestant	31	34.8
**Total**	89	100

**Clinical characteristics of patients:** Table 3 shows that the breast most affected by cancer was the right breast, with 54.25% (115 out of 212), and 87.74% (186 out of 212) of patients had invasive ductal carcinoma, of whom 102 (48.34%) were stage II, and 54.72% (116 out of 212) grade 2. In this study, 51.67% (111 out of 212) of patients had received chemotherapy. In the prostate cancer population (Table 4), the most common circumstance of discovery was mictional disorder, with 52 out of 89 (58.43%). The most common associated pathology was pollakiuria, with 30/89 (33.71%), and the most frequent type of sampling was biopsy, with 61 (68.54%), and 25 (28.09%) out of 89 transurethral resections of the prostate (Table 4).

**Table 3:** clinical characteristics of patients with breast cancer

Variables	Frequency	Percentage (%)
**Mammary pain**		
Yes	115	54.2
No	97	45.8
**Breast discharge**		
No	164	77.4
Yes	48	22.6
**Breast affected**		
Right	115	54.2
Left	97	45.8
**Histological type**		
IDC	186	87.7
LDC	26	12.3
**AJCC Stade (n=211)**		
0	1	0.5
I	51	24.2
II	102	48.3
III	40	19.0
IV	17	8.1
**Grade SBR**		
1	44	20.8
2	116	54.7
3	52	24.5
**Chemotherapy before**		
Yes	111	51.7
No	101	48.3
**Total**	212	100

AJCC: American Joint Committee on Cancer; IDC: invasive ductal carcinoma; LDC: lobular ductal carcinoma; SBR: Scarff-Bloom-Richardson

**Table 4:** clinical characteristics of patients with prostate cancer

Variables	Frequency	Percentage (%)
**Circumstances of discovery**		
Asthenia	2	2.2
Consultation	8	9.0
Erectile dysfunction	27	30.3
Urination disorders	52	58.4
**Associated diseases**		
Cystitis	8	9.0
Oedema	1	1.1
Pollakiuria	30	33.7
Prostatitis	10	11.2
Pyelonephritis	19	21.4
Uretritis	21	23.6
**Nature of the sample**		
Biopsy	61	68.5
Operating room	3	3.4
Rtup	25	28.1
**Total**	89	100

**Factors associated with survival:** Table 5 shows that more than half of the total population of BCa patients had TILs, and the high grade of TILs was the most represented with 36.32% (77 out of 212) in this population. While in PCa, the most represented grade of TILs was moderate grade with a percentage of 31.46% (28/89) TILs, followed by low grade with 25.84% (23/89) TILs in the overall population (Table 5). Table 6 depicts the association between TILs grade and chemotherapy in the BCa population. It can be observed that the intermediate TILs grade had an odds ratio > 1, indicative of a positive association between the two variables. Intermediate TILs grade was positively associated with a good response to chemotherapy (p < 0.05). In contrast, in PCa, low TILs grade had an odds ratio> 1, indicating a positive link between the two variables (p< 0.05), suggesting that in both BCa and PCa, TILs grade is proportionally associated with therapy (Table 6).

**Table 5:** distribution of TILs grades in breast and prostate cancer patient populations

Grade TILS	Frequency	Percentage (%)
**Breast cancer**		
Absent	37	17.5
Low (0-11%)	39	18.4
Moderate (11-59%)	59	27.8
High (≥ 60%)	77	36.3
**Total**	212	100.00
**Prostate cancer**		
Absent	33	37.1
Low (0-11%)	23	25.8
Moderate (11-59%)	28	31.5
High (≥60%)	5	5.6
**Total**	89	100

TILs: tumor-infiltrating lymphocytes

**Table 6:** association between TILs grade and chemotherapy for breast cancer

Breast cancer						
Grade TILs	N	Chemotherapy		OR	[95% CI]	p-value
		Yes	No			
Absent	37	17	20	0.85	0.44-1.62	0.6223
Low	39	14	25	0.56	0.29-1.07	0.0824
Moderate	59	38	21	1.8095	1.06-3.08	0.0292
High	77	42	35	1.2	0.76-1.87	0.4257
**Prostate cancer**						
**Grade TILs**	**N**	**Hormone-therapy**		**OR**	**[95% CI]**	**p-value**
		**Yes**	**No**			
Absent	33	14	19	0.7368	0.4-1.5	0.3859
Low	23	17	6	2.8333	1.11-7.18	0.0283
Moderate	28	11	17	0.6471	0.30-1.38	0.2606
High	5	4	1	0.25	0.028-2.23	0.215


TILs: tumor-infiltrating lymphocytes; OR: odds ratio; CI: confidence interval

**Figure 2:**
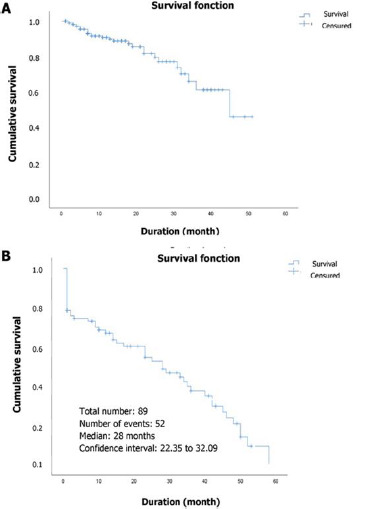
distribution of overall survival in breast (A) and prostate (B) cancer patients

**Figure 3:**
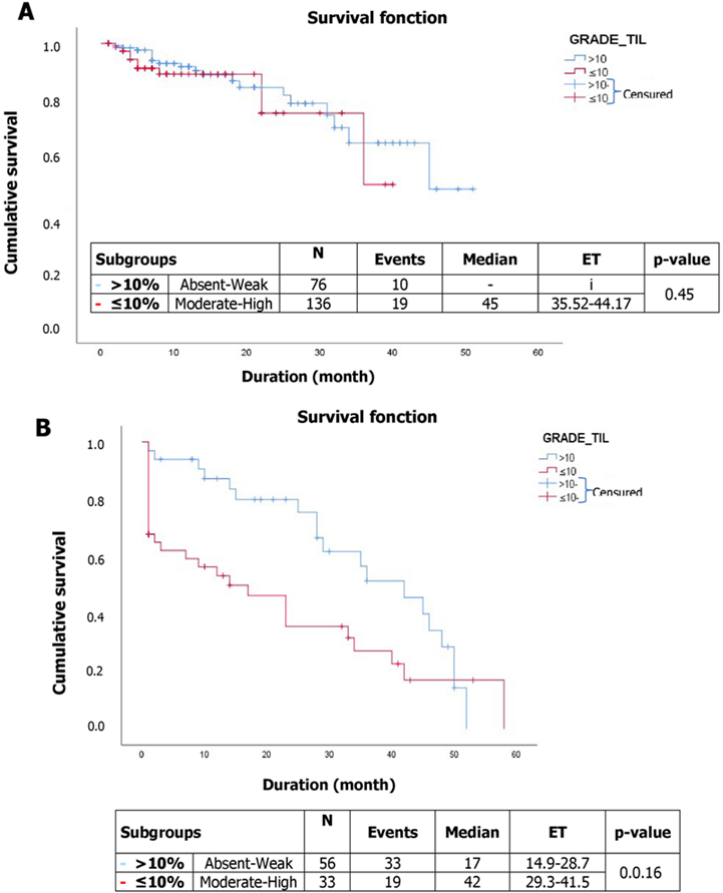
distribution of the association between tumor-infiltrating lymphocytes (TIL) grade and overall survival time in breast (A) and prostate (B) cancer patients

**Correlation between TILs grades and 5-year overall survival:** Figure 2A shows through the Kaplan-Meier curve the distribution of survival time in the BCa population. A total of 29 events were recorded in the population with a median survival of 45 months, and a confidence interval (95% CI) of 35.14 to 42.89. Overall survival was estimated at 44% at 51 months. Similarly, 89 patients had prostate cancer with 52 deaths recorded with a median survival of 28 months [95% CI = 22.348 to 32.091] and overall survival of 59 months. Overall survival for prostate cancer estimated by the Kaplan-Meier method is presented in Figure 2B. Figure 3A presents an association between TILs subgroups and survival time in the BCa population. Indeed, 10 events were recorded in the absent-low TIL grade subgroups, and the median was not reached. In contrast, those with intermediate-high TILs grade had 19 events with a median survival of 45 months [95% CI= 35.52 - 44.17]. No positive correlation was found between TILs grade and survival in BCa patients (p= 0.45) (Table 7). The correlation between TILs grade and 5-year overall survival shows that the absent-low TILs subgroup had a median survival of 17 months, and the intermediate-high TILs subgroup had a median survival of 42 months, with a p-value of 0.016, which is statistically significant (Figure 3B). Patients with prostate cancer who had not taken treatment had a 7.23 greater risk of death (HR: 7.23, 95% CI 1.21-53.14; p= 0.049) than those who had (Table 7).

**Table 7:** risk factors for survival in patients with breast cancer and prostate cancer

Breast cancer	HR	[95% CI]	p-value
**Lymphocyte infiltration**			
No	1		
Yes	0.60	0.31-1.15	0.127
**Grade of TILs**			
Absent	1		
Weak	1.00	0.42-2.41	0.992
Moderate	1.30	0.42-4.00	0.650
High	0.52	0.17-1.64	0.267
**Age**			
20-35	1		
35-50	0.74	0.37-1.48	0.398
50-65	0.94	0.45-2.09	0.942
65-75	0.27	0.03-2.15	0.218
**AJCC stage**			
0	1		
I	1.59	0.50-5.07	0.429
II	1.46	0.50-4.25	0.375
III	1.62	0.49-5.34	0.425
IV	1		
**Treatment**			
No	1		
Yes	1.20	0.68-2.14	
**Surgery**			
No	1		
Yes	1.70	0.80-3.59	0.168
**Prostate cancer**	**HR**	**[95% CI]**	**p-value**
**Lymphocyte infiltration**			
No	1		
Yes	1.13	0.58-2.19	0.725
**Grade of TILs**			
Absent	1		
Weak	1.06	0.48-2.32	0.891
Moderate	1.12	0.50-2.53	0.78
High	1.44	0.46-4.48	0.526
**Age**			
<65	1		
65-74	1.17	0.40-3.46	0.776
75-84	1.78	0.66-4.45	0.255
85+	2.16	0.68-6.84	0.189
**AJCC stage**			
IIA	1		
IIB	2.33	0.68-7.95	0.178
IVA	2.12	0.54-8.22	0.279
IVB	2.22	0.62-7.96	0.219
**Treatment**			
No	1		
Yes	7.23	1.2-53.14	**0.049**

AJCC: American Joint Committee on Cancer; TILs: tumor-infiltrating lymphocytes; CI: confidence interval; HR: hazard ratio

## Discussion

According to the estimation of the WHO, there will be 21.7 million new cases of cancer and 13 million deaths in Africa by 2030, due to the increase in life expectancy worldwide, if nothing is done [13]. Cameroon, like other developing countries, is faced with insufficient resources to ensure adequate care for patients with cancer, which is on the increase. Moreover, cancer is a public health problem that must be considered in light of the great morbidity it inflicts on patients. Tumor-infiltrating lymphocytes (TILs) are a heterogeneous group of cells comprising B cells, T cells, and natural killer (NK) cells that migrated from the bloodstream to a tumor and are found both in the tumor itself and in the stroma surrounding it [9,10]. They are considered a good prognostic marker for cancers as they can indicate the body's immune response to the tumor and are responsible for the development of antitumor immune responses which can detect tumor antigens and destroy them [11]. Despite this, no studies correlating TILs with the survival of patients with hormone-sensitive (breast and prostate) cancers have been conducted in Cameroon. The purpose of this study was therefore to evaluate the relationship between TILs and survival in patients with breast and prostate cancers over the last 5 years at YGH.

The results obtained from 212 BCa patients showed that the mean age was 43.56 ± 11.90 years. These results are similar to those of Fouhi *et al*. [4], who also found an average age of 47 ± 12 years in Casablanca (Morocco) and those of Essiben *et al*. [14] carried out at the YGH and YGOPH who found an average age of 42 ± 12.1 years. This could be explained by the fact, as shown by Saglier *et al*. [1], that breast cancer has a high incidence in people aged around 50. We also found that 77.83% (165 out of 212) of this population were in menopause. These results are similar to those of Fejzić *et al*. [15], who found that 53.33% of patients were menopausal. In fact, menopause is a genetically programmed phenomenon that occurs on average at the age of 50. Menopause is well known as a risk factor for hormone-sensitive breast cancers, particularly because of the hormonal changes that occur at this time, such as the drop in estrogen levels.

In the population of prostate cancer patients, the age group most represented was that between 75 and 84. These results are in line with those of the World Cancer Observatory, which shows a high incidence in people aged over 70 [3]. This implies that in Cameroon, as elsewhere, older men were the most affected. The majority of patients discovered their pathology by a mictional disorder with 52 (58.43%) cases. The associated pathology was pollakiuria in 30 (33.71%) cases. These results are in line with the recommendations of Rozet *et al*. [16], which states that signs and symptoms often appear as the tumor develops and that urinary and erectile disorders are part of the changes that the human body can undergo at a certain age.

A total of 82.55% (175 out of 212) of patients had TILs. These results corroborate those of Huszno *et al*. [17], who found that 87% of BCa patients had TILs. All this reflects the body's immune response against the tumor. Lymphocytes are immune system cells that can recognize and destroy tumor cells. Determination of the percentage of TILs revealed four TILs grades in our study population: absent TIL grade (17.45%), low TIL grade (18.40%), intermediate TIL grade (27.83%), and high TIL grade (36.32%). These results are similar to those of Ono *et al*. [18], who worked on 180 patients with triple-negative breast cancer and found that high (34%) and low (19%) TILs grades were the most represented.

Of the 89 patients with prostate cancer, 57 (64.04%) patients had TILs, 28 (31.46%) of cases were of moderate TILs grade, followed by 23 (25.84%) of low TILs grade regarding tumor extension. These results differ from those of Vilaça *et al*. [19], who found a high infiltration of TILs (41/31.87%/63%) on tumor-infiltrating lymphocytes in localized prostate cancer in a study of 96 patients. Low-grade TILs were significantly associated with hormone therapy, with an odds ratio of 2.83 [95% CI= 1.11-7.18] and a p-value of 0.0283. These results are in agreement with those of Bhinder *et al*. [20], where low infiltration associated with hormone therapy presented low ratios in lethal PCa.

In 2021, Zingue *et al*. [7] reported that the 5-year survival rate for BCa in Cameroon was 43.3%. In our BCa patients, using the Kaplan-Meier curve, the 5-year overall survival was estimated at 44% with a maximum number of months of equal survival of 51 months. No statistically significant association was found between 5-year survival and the different subgroups of TILs constituted: absent-low TILs and intermediate-high TILs (p= 0.45). According to Romagnoli *et al*. [21], TIL-rich tumors predict overall survival. The fact that we did not observe an association between TILs grade and 5-year survival in this population may be due to the fact that not all molecular subtypes of breast cancer were considered separately.

The median 5-year survival in the PCa population was estimated at 28 months, with patients with very little infiltration showing a significantly improved survival of 17 months, and those with moderate to high infiltration showing a significantly improved survival of 42 months. There was also a gain in survival at 25 months with a p-value of 0.016. These results corroborate the observations made by Vilaça *et al*. [19], whose show that TILs in localized PCa was statistically significant (p < 0.05) and appear to be an important prognostic variable for recurrence of localized prostate cancer.

## Conclusion

The mean age was 43.56 ± 11.90 years for BCa with stage II and SBR grade II being the most represented. While in Pca, the mean age was 75 to 84 years (34.83%) with stage II being the most frequent. Four TILs grades: absent (17.45%), low (18.40%), intermediate (27.83%) and high TIL grade (36.32%) were noted in BCa, while in PCa the most represented TILs grade was intermediate (31.46%). In BCa the intermediate TILs grade was positively associated with a good response to chemotherapy (p< 0.05). The overall 5-year survival was around 44% for BCa wheras it was 42 months for those with infiltration in PCa. No positive correlation was found between TILs grade and survival in BCa patients (p= 0.45), while in PCa it was found that TILs is significantly correlated (p= 0.016) with the survival. Further studies are encouraged to investigate the association between BCa molecular groups and TILs.

### What is known about this topic

- It is well known that early diagnosis of cancer allows effective treatment at lower cost and improves the overall survival of patients;
- Cameroonian authors reported that 5-year overall survival in Cameroon was around 43.3% between 2010-2015;
- The presence of TILs in breast and prostate cancers is associated with a good response to treatments (chemotherapy or hormone therapy) and with the survival.

### What this study adds

- This study provides new data on the characteristics of patients suffering from breast and prostate cancers in Cameroon;
- Positive association was found between intermediate-TILs grade and response to chemotherapy (p< 0.05), and low-TILs grade and hormone therapy (p= 0.0283) for prostate cancer;
- Five-year survival in Cameroon between 2019 and 2023 was 44% for breast cancer and 0% for prostate cancer, showing that efforts are being made to combat cancer.
